# Between Hearts and Gears: Technology at the Service of Life

**DOI:** 10.21470/1678-9741-2025-0167

**Published:** 2025-12-10

**Authors:** Paulo Cesar Duarte Junior, Alexandre Noboru Murakami, Aron José Pazin de Andrade

**Affiliations:** 1 Department of Bioengineering, Instituto Dante Pazzanese de Cardiologia, São Paulo, São Paulo, Brazil paulo.duarte@dantepazzanese.org.br; 2 Department of Civil Construction, Universidade Estadual de Londrina, Londrina, Paraná, Brazil; 3 Department of Surgical Clinics, Universidade Estadual de Londrina, Londrina, Paraná, Brazil

This letter is an invitation to reflect on the role of technology and interdisciplinary collaboration in addressing the challenges posed by congenital heart disease. It is not a defense of engineering, nor a glorification of technology. It is about recognizing that these tools, when guided by human and ethical purposes, have the potential to transform realities, especially those marked by anatomical complexity and clinical urgency.

For decades, medicine has continuously reinvented itself. New surgical techniques, diagnostic advances, and safer therapies are part of this trajectory^[1]^. In parallel, other sciences such as engineering have quietly approached the field. Not with the intention of taking the place of the surgeon, but with a willingness to support, through simulations that anticipate scenarios, devices designed with precision, and three-dimensional representations capable of revealing what cannot be seen with the naked eye^[2]^.

What has become common in industry, such as the use of robots to optimize production lines, is still developing in healthcare^[3]^. Interestingly, in those sectors, automation has not reduced jobs. On the contrary, it has redefined roles, increased safety, and enhanced efficiency. Why not envision something similar in the hospital setting?

The intention is not to elevate one science above another, but to build bridges. Engineering alone does not save lives. Medicine, by itself, continues to save many, but often at the cost of immense human and structural effort. True transformation arises from the intersection of knowledge. From continuous, institutionalized integration between specialties that, together, can anticipate problems, propose solutions, and expand access.

Today, we can already envision a future where algorithms help to identify vascular geometries, simulate hemodynamics, predict risks, and suggest personalized treatments^[4]^. This is no longer science fiction. It is becoming a reality in centers that have chosen to integrate technical knowledge into clinical care. What we still lack is not technology, but vision. Vision to incorporate these tools critically, ethically, and with a commitment to equity.

Above all, it is about broadening access. Planning technologies, innovative materials, predictive models, and simulation-based training should not be exclusive instruments, but tools that promote justice in healthcare. The true impact of innovation occurs when it reaches those who need it most, shortening the distance between diagnosis and intervention, between technical knowledge and family relief.

A few months ago, a study based on three-dimensional numerical simulations applied to the Fontan procedure was published in this journal^[2]^. More recently, in Medical Engineering & Physics, this approach was further developed through a multiparametric analysis of the total cavopulmonary connection^[5]^. Additionally, artificial intelligence was used to map critical gaps in the literature on pediatric implantable devices, revealing neglected areas^[6]^. These examples demonstrate that integration between engineers and physicians is not just desirable, it is necessary.

Such technologies do not aim to replace human hands, but to strengthen them. They do not intend to dictate conduct, but to support decision-making. They represent a way to multiply accumulated knowledge, to train new professionals in safe environments, and to improve surgical outcomes through robust preoperative analysis.

No surgery is performed in isolation. There is always a team. Perhaps it is time to broaden the meaning of that word, including specialists from other areas: computer scientists, biomedical engineers, mathematicians, physicists. Not to interfere with clinical judgment, but to contribute to its preparation, safety, and effectiveness. Genius today does not lie in solitary specialization, but in the solidarity of intersecting worlds.

We would like to conclude with an image: a silent operating room, surrounded by screens displaying flows, pressures, geometries, and probabilities. At the center, a dedicated team, making decisions based on data, but guided, above all, by an unwavering conviction, that every heartbeat is a universe worth protecting. May technology never lead us to forget this essential truth.

## Artificial Intelligence Usage

The authors declare the use of ChatGPT (OpenAI) solely for language editing and text refinement. All scientific content, including conception, methodology, results, and interpretations, is the authors’ own work. The final manuscript was thoroughly revised and validated by the authors, who take full responsibility for the published content.
